# Familial multinodular goiter syndrome with papillary thyroid carcinomas: mutational analysis of the associated genes in 5 cases from 1 Chinese family

**DOI:** 10.1186/1472-6823-13-48

**Published:** 2013-10-21

**Authors:** Shunyao Liao, Wenzhong Song, Yunqiang Liu, Shaoping Deng, Yaming Liang, Zhenlin Tang, Jiyuan Huang, Dandan Dong, Gang Xu

**Affiliations:** 1 Diabetes & Endocrinology Center, Sichuan Academy of Medical Science, Sichuan Provincial People’s Hospital, Chengdu 610072, China; 2 Department of Thyroid Disease & Nuclear Medicine, Sichuan Academy of Medical Science, Sichuan Provincial People’s Hospital, Chengdu 610072, China; 3 Department of Medical Genetics and Division of Morbid Genomics, State Key Laboratory of Biotherapy, West China Hospital, Sichuan University, Chengdu 610041, China; 4 Department of Surgery, Harvard Medical School, Massachusetts General Hospital, Boston, MA, USA; 5 Department of Pathology, Sichuan Academy of Medical Science, Sichuan Provincial People’s Hospital, Chengdu 610072, China

**Keywords:** Familial papillary thyroid carcinomas, Multinodular goiter syndrome, Mutational analysis, Genetic association, Risk alleles

## Abstract

**Background:**

Familial papillary thyroid cancer (fPTC) is recognized as a distinct entity only recently and no fPTC predisposing genes have been identified. Several potential regions and susceptibility loci for sporadic PTC have been reported. We aimed to evaluate the role of the reported susceptibility loci and potential risk genomic region in a Chinese familial multinodular goiter (fMNG) with PTC family.

**Methods:**

We sequenced the related risk genomic regions and analyzed the known PTC susceptibility loci in the Chinese family members who consented to join the study. These loci included (1) the point mutations of the *BRAF* and *RET*; (2) the possible susceptibility loci to sporadic PTC; and (3) the suggested potential fMNG syndrome with PTC risk region.

**Results:**

The members showed no mutations in the common susceptible *BRAF* and *RET* genomic region, although contained several different heterozygous alleles in the *RET* introns. All the members were homozygous for PTC risk alleles of rs966423 (C) at chromosome 2q35, rs2910164 (C) at chromosome 5q24 and rs2439302 (G) at chromosome 8p12; while carried no risk allele of rs4733616 (T) at chromosome 8q24, rs965513 (A) or rs1867277 (A) at chromosome 9q22 which were associated with radiation-related PTC. The frequency of the risk allele of rs944289 (T) but not that of rs116909374 (T) at chromosome 14q13 was increased in the MNG or PTC family members.

**Conclusions:**

Our work provided additional evidence to the genetic predisposition to a Chinese familial form of MNG with PTC. The family members carried quite a few risk alleles found in sporadic PTC; particularly, homozygous rs944289 (T) at chromosome 14q13 which was previously shown to be linked to a form of fMNG with PTC. Moreover, the genetic determinants of radiation-related PTC were not presented in this family.

## Background

PTC is the most prevalent malignancy of the thyroid gland. There has been an increasing incidence of PTC worldwide for the past few decades. The etiology of PTC is related to environmental, hormonal and genetic factors. About 5-15% of PTC patients show a familial occurrence, and fPTC is recognized as a distinct entity only in recent years [1,2]. Families with accumulation of PTCs show an inherited trait of the disease and patients with fPTC often have early age at disease onset and increased severity in successive generations, also, fPTC patients frequently present more aggressive tumors with increased incidence of multifocality, local invasion, lymph node metastases than the sporadic PTC [2,3]. Generally, fPTC is diagnosed when three or more family members have PTC and in the absence of other known associated syndromes [1,2]. PTC has a significant gender bias with much more women affected than men; it is especially suggestive for the familial predisposition when men or children were diagnosed with PTC [1,4]. While, because families share the same environment and a common genetic background, it is difficult to distinguish between environmental and genetic contributing factors, and also because the majority of fPTC pedigrees are small in size and may present with a variety of additional benign thyroid nodules, the genetic predisposition to fPTC is unknown and the molecular alterations at the origin of the pathology are only now beginning to emerge [1,5,6].

Sporadic PTC is known to be associated with point mutation of the *BRAF* genes and chromosomal rearrangements of *RET*/*PTC*. The *BRAF* encodes a serine/threonine-protein kinase which plays a role in regulating the MAP kinase/ERKs signaling pathway and affects cell division, differentiation and secretion; point mutations in *BRAF* are found in up to 45% PTC cases [7]. The *RET* protooncogene is one of the receptor tyrosine kinases, cell-surface molecules that transduce signals for cell growth and differentiation; rearrangements of the *RET* are found in about 35% of sporadic PTC [7]. Although somatic mutations of the genes like *BRAF* and *RET* exclusively play a causative role in sporadic thyroid cancer development, germline mutations of single nucleotide polymorphisms (SNPs) in these genes were also reported to act as modifiers in the cancer process [8,9], it needs to mention here that in a Chinese population, SNPs of *BRAF* were shown to be associated with PTC [10], and thus it is intriguing to verify these mutations in fPTC families.

Recent studies based on population stratification have made progresses to identify several single nucleotide polymorphisms (SNPs) associated with PTC risk. For examples, (1) It was discovered that rs966423 at 2q35, locating into the intron region of the disrupted in renal carcinoma 3 gene (*DIRC3*), was significantly associate with European nonmedullary thyroid cancer (NMTC) by the genome-wide studies [11]. *DIRC3* predicted a non coding RNA transcript with unknown function, the first 2 exons of *DIRC3* replaced exon 1 of *HSPBAP1* and formed a DIRC3-HSPBAP1 fusion transcript, which are associated with chromatin remodeling and stress response; (2) It was reported that the heterozygosity G/C of SNP rs2910164 at 5q24 within the precursor of *microRNA-146a* predisposed to PTC by altering expression of miR146a target genes in the Toll-like receptor and cytokine signaling pathway [12,13]; (3) The genome-wide study also identified that chromosomal 8q24 was associated with the risk of various cancers, particularly, rs4733616 at 8q24 was founded to be possibly associated with PTC risk in 26 European families [14-16]; (4) The rs2439302, located in the intron of *HRG-beta1c* at 8p12,was reported to be associated with *neuregulin 1* (*NRG1*) and confer risk of thyroid cancer [11]. HRG-beta1c is one of the NRG1 isoforms and interacts with tyrosine kinase to increase its phosphorylation on tyrosine residues, playing critical roles in the growth and development of multiple organ system; (5) It was repeatedly observed that the rs965513 at 9q22.33 were the strong association signal for NMTC in European people [16-19] and it was proposed that the rs965513 might linked to the nearest thyroid transcription factor of forkhead family (*FOXE1*) gene, which likely plays a crucial role in thyroid morphogenesis; furthermore, some research indicated that rs1867277 within the *FOXE1* 5′ UTR is also a causal variant in thyroid cancer susceptibility [16,20]; (6) Finally, both rs944289 and rs116909374 on 14q13.3 were observed to be strongly associated with NMTC in European people [11,16-19,21]. Nonetheless, all these genetic associations found by the genome-wide association studies have not been investigated in a family based study.

In addition, a few potential regions for harboring an fPTC gene have been reported: chromosomal region 1q21 linked to fPTC with papillary renal neoplasia [22], 2q21 linked to familial NMTC type 1 syndrome [23], and the telomere abnormalities and chromosome fragility might display in fPTC family [24]; Specifically, familial NMTC and its relationship with familial MNG are recognized as distinct clinical entities, and the molecular pathophysiology of MNG and PTC is different, indeed MNG1 is located at 14q [25]; however, one study in a kindred with MNG and PTC suggested that 14q32 linked to a form of inherited MNG syndrome with a significant risk of progression to PTC [26].

**Figure 1:**
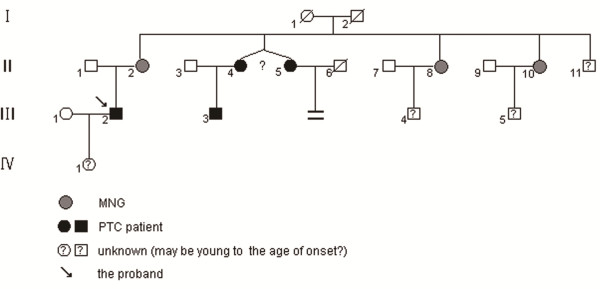
**Pedigree of the Chinese fPTC.** Circles and squares indicate female and male family members, respectively. The proband is indicated by an arrow.

In the present report we studied 2 PTCs and 3 MNGs obtained from members of one Chinese family. This family was ascertained through initial identification of the proband, a 35-year-old men (III2, Figure 1). The probands’s mother, 5 maternal aunts and 1 younger first cousin were diagnosed with MNG or PTC by different hospitals in China. The mode of inheritance in the family appeared to be autosomal dominant. For the purpose to improve our understanding of the PTC predisposition, based on the recent progresses in genetic studies about PTC, we analyzed in this Chinese family (1) the point mutations of the *BRAF* and *RET*; (2) the possible susceptibility loci to sporadic PTC; and (3) the suggested potential fMNG syndrome with PTC risk region.

## Methods

### Patients

The fMNG with PTC pedigree is reported in Figure 1. The clinical and pathological findings are summarized in Table 1.

**Table 1:** Clinical and pathological study of the collected samples

**Members**	**Sex**	**Age at diagnosis**	**Histology**	**sizes for PTCs and MNGs**	**Surgical treatment**
II1	male	64	normal		
II2	female	62	bilateral MNG	MNG (1.2 cm), suspicious lesion	completion thyroidectomy
III2	male	35	bilateral MNG with PTC	PTC in MNG, PTC (1.6 cm)	completion thyroidectomy
II5	female	56	bilateral MNG with PTC	PTC in MNG, PTC (1.5 cm)	completion thyroidectomy
II8	female	45	bilateral MNG	MNG (0.3 cm)	
II10	female	41	MNG in right thyroid	MNG (0.6 cm)	

The study protocol was approved by the Review Board of Clinical Research of the Sichuan Provincial hospital, and by the Research & Ethics Committee of Sichuan Medical Research Institution. The blood samples were collected from the proband (III2), proband’s parents (II1 & 2) and maternal aunts (II5, 8, & 10) with their written informed consent.

**Figure 2:**
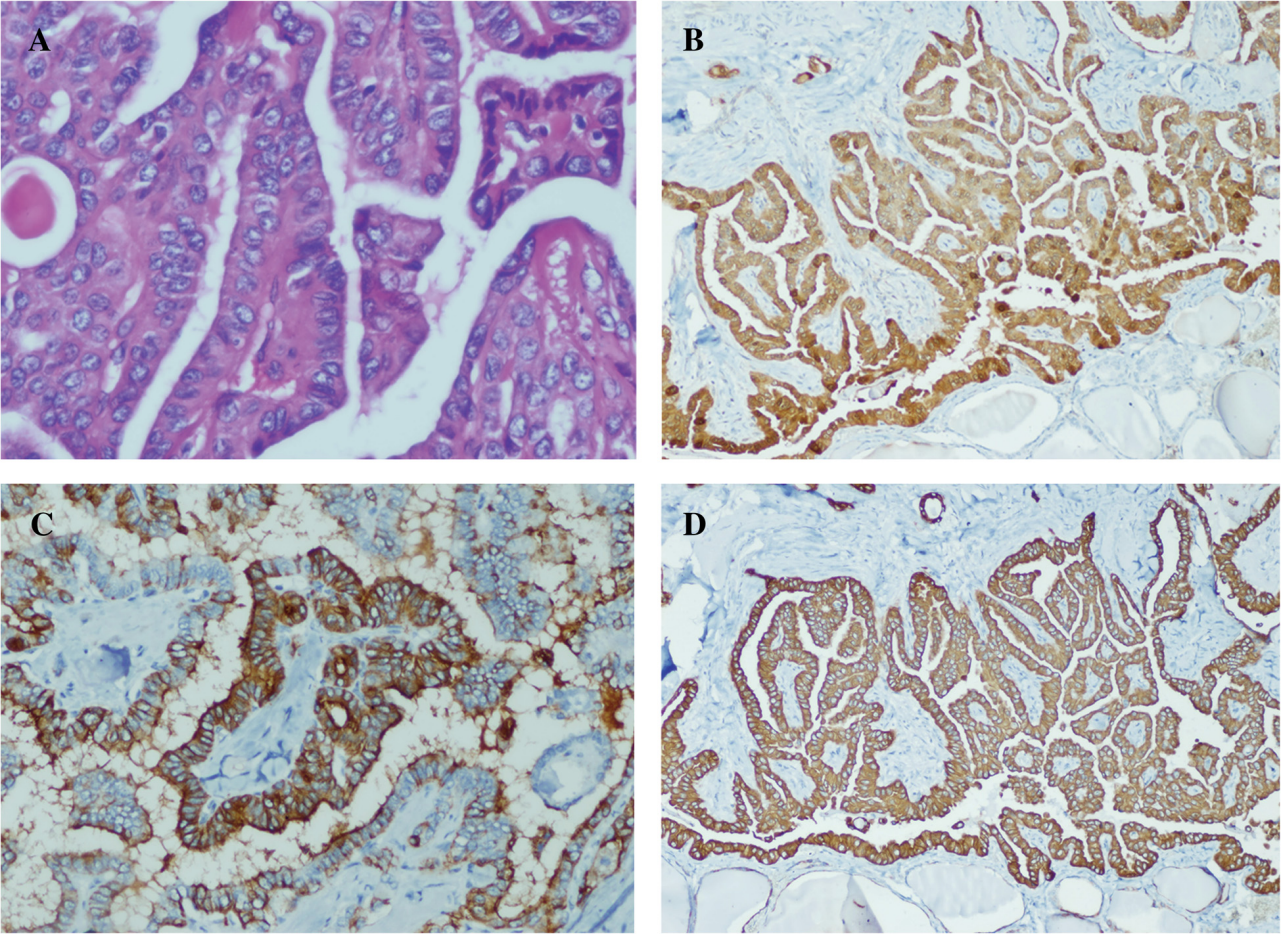
**The histological features of the proband’s papillary carcinoma. A**: The cytological feature: crowded oval nuclei, nuclear grooves, clearing, elongation and overlapping (HE × 400). **B**: Galectin-3 showed predominantly cytoplasmic staining with occasional nuclear staining (×200). **C**: HBME1 showed positive diffuse membrane (×200). **D**: Cytokeratin 19 showed strong, predominantly cytoplasmic staining (×200).

A 35-year-old man (Figure 1 III2) came to our observation: the man complained both his lymph nodes containing palpable lump for more than 10 days, initial ultrasound examinations revealed an 1.9 × 1.4 cm solid mass with irregular & indefinite border, sand calcification and blood flow in his right neck, and also 2 small nodule goiters in his left neck; The thyroid function tests showed the man was euthyroid; both the fine needle aspiration cytological and thyroidectomy specimen pathologic examinations disclosed that the architecture and nuclear features of the neoplasm in his both necks were typical for PTC (Figure 2A) and immunohistochemical staining confirmed the diagnosis (Figure 2B, C, D); After the total thyroidectomy and radioactive iodine treatment, the patient is now doing well. Interestingly, in terms of fMNG with PTC, the patient’s mother is diagnosed with MNG in bilateral thyroid and underwent a total thyroidectomy in Chongqing, China (Figure 1 II2). Both of the patient’s maternal twin aunts and a younger male cousin were diagnosed with MNG and PTC by different hospitals in Beijing and Chongqing, China, respectively; the other two maternal aunts were diagnosed with MNG by different hospitals in Chengdu and Dazhou, China, respectively (Figure 1 II8&10).

### DNA extraction

The whole blood was collected from the medial cubital vein into heparin anticoagulant tubes. The total DNA was purified using the spin protocol of QIAamp DNA Blood Mini Kit according to the manufacturer’s directions (Qiagen, Hilden, Germany). The purified DNA was resuspended in TE buffer and stored at 4 °C. Gel electrophoresis and spectrophotometric determination were used to DNA quantification and quality analysis. The OD260/OD280 ratio of DNA samples were between 1.8-2.0 and concentration was more than 100 ng/ml.

### Genetic mutational analysis

The potential regions and susceptibility loci investigated in the study were listed in Table 2. Sequencing was performed on PCR-amplified products using primers (Table 2) according to the published sequences or self-designed with Primer Premier 6.1 (PREMIER Biosoft, Palo Alto CA). The PCR amplifications were performed using ABI GeneAmp PCR System 9700 (Applied Biosystems, Foster City, CA). The PCR reaction system included 2U *Pfu* DNA polymerase (Thermo Fisher Scientific Inc, USA), 50pmol of each sense and antisense primers, 1 × reaction buffer (20 mM Tris–HCl pH8.8, 10 mM KCl, 10 mM (NH4)_2_SO_4_, 1% (v/v) Triton X-100), 250 μM dNTP, 2.0 mM MgCl_2_ and 200 ng genomic DNA in a total volume of 50 μl. The PCR cycling parameters were followed the recommendations for *Pfu* DNA polymerase according to the manufacturer. Precautions were taken to prevent PCR contamination, and indeed, in each experiment DNA template negative samples were run in parallel. The PCR products were resolved by electrophoresis in a 2% agarose gel stained with ethidium bromide and purifed using the QIAquick PCR purification kit (Qiagen). Purified PCR products were sequenced directly in both orientations using standard procedures with an ABI PRISM 3100 Genetic Analyzer (ABI, CA). The sequences were confirmed with two independent PCRs from two independent DNA samples.

**Table 2:** Sequences of the primers

**Clinical channel**	**Primers**	**Localization & product**
*BRAF* at Chr7q34: 140,433,812-140,624,564(190,752 bp)		
exon15: 176,372-176,490 (119 bp) K601E: 176,431(A → G) rs121913364: 140,453,134 V600E: 176,429 (T → A) rs113488022: 140,453,136	5′-TGCTTGCTCTGATAGGAAAATG-3′ 5′-CCACAAAATGGATCCAGACA-3′	Chr7:140,453,250-140,453,078 (173 bp) intron: 176,315-176,371 exon15:176,372-176,487 (116 bp)
*RET* at Chr10q11.2: 43,572,517-43,625,799		
exon5: 34,308-34,503 (196 bp) R313Q: 34,378(G → A) rs77702891: 43,601,894 R330Q: 34,429(G → A) rs80236571: 43,601,945	5′-CTTTCCTCACAACCCCCTCC-3′ 5′-AGAGCGAGCACCTCATTTCC-3′	Chr10: 43,601,341-43,602, 077 (737 bp) intron: 33, 825–34,307&34,504-34,561 exon5: 34,308-34,503 (196 bp) STS: 33,825-34,398
exon8: 40,031-40,156 (126 bp) G533C: 40,105(G → T) rs75873440: 43,607,621	5′-CCTGTGCAGTCAGCAAGAGA-3′ 5′-CCTGTTCCCATGCCCTGATT-3′	Chr10: 43,607,577-43,608,444 (868 bp) exon8: 40,061-40,155 (96 bp) intron: 40,156-40,784&40,896-40,928 exon9: 40,785-40,895 (111 bp)
exon10: 41,488-41,607(120 bp) C609R: 41,553(T → C) rs77558292: 43,609,069 C609Y: 41,554(G → A) rs77939446: 43,609,070 C611W: 41,561 (C → G) rs80069458: 43,609,077 C618R/G: 41,580(T → C/G) rs76262710: 43,609,096 C618S: 41,581(G → C) rs79781594: 43,609,097 C620R: 41,586(T → C) rs77316810: 43,609,102 C620F/S/Y: 41,587(G → A/C/T) rs77503355: 43,609,103 C620W: 41,588(C → G) rs79890926: 43,609,104 cds-indel: 41,562_41,588del27 rs121913313: 43,609,078_43,609,104del27	5′-GGAAACCTGGATCCCACAGG-3′ 5′-GGGAGGGAAGTTTCATGGGG-3′	Chr10: 43,608,459-43,609,249 (791 bp) intron: 40,943-41,487&41,608-41,557 exon10: 41,488-41,607(120 bp) STS: 41558-41733
exon12: 44,516-44,663 (148 bp)	5′-GTGGGCCCAATGTGTGGATA -3′ 5′-CTCTTCAGGGTCCCATGCTG-3′	Chr10: 43,611,512-43,612,272 (761 bp) intron: 43,996-44,515&44,664-44,756 exon10: 44,516-44,663 (148 bp)
exon13: 46, 305–46,412 (108 bp) S765P: 46,313(T → C) rs75075748: 43,613,829 E768E: 46,324(G → A/C) rs78014899: 43,613,840 V778I: 46,352(G → A) rs75686697: 43,613,868 L790F: 46,390(G → C) rs75030001: 43,613,906 Y791F: 46,392(A → T) rs77724903: 43,613,908	5′-CGGGGAATTTCTGTGGACGA-3′ 5′-ATGGCAGTGTCACACCAGAG-3′	Chr10: 43,613,496-43,614,200 (705 bp) intron: 45,980-46,304&46,413-46,684 exon13: 46, 305–46,412 (108 bp) misc_difference: 46,327
exon14: 47, 463–47,677 (215 bp) V804M: 47,480(G → A/T) rs79658334: 43,614, 996	5′-GAGGCAGAGAGCAAGTGGTT-3′ 5′-AATAGCACGAGTCGTCAGGC-3′	Chr10: 43,614,767-43,615,517 (751 bp) intron: 47,251-47, 462&47,678-48,001 exon14: 47, 463–47,677 (215 bp)
exon15: 48,013-48,135 (123 bp) S891A: 48,076(T → G) rs75234356: 43,615,592 cds-indel: 48,051_48,053delAGCinsTTT rs121913306 43,615,567_43,615,567delins R897Q: 48,095(G → A) rs76087194: 43,615,611 cds-indel: 48,097_48,108del12 rs121913309: 43,615,613_43,615,624del12	5′-TCTCACAGGGGATGCAGTATCTG-3′ 5′-GAGGCTGAGCGGAGTTCTAATTG-3′	Chr10: 43,615,159-43,615,837 (679 bp) exon14: 47,643-47,677 (35 bp) intron: 47,678-48,012&48,136-48,321 exon15: 48,013-48,135 (123 bp)
exon16: 49, 878–49,948 (71 bp) M918T: 49,900(T → C) rs74799832: 43,617,416 R912P: 49,882(G → C/T) rs78347871: 43,617,398	5′-GCTCCAGCCCCTTCAAAGAT-3′5′-CTTTGAGCAGTTTGGGGCAC-3′	Chr10: 43,617,229-43,617,941 (713 bp) intron: 49,713-49, 877&49, 949–50,425 exon16: 49,878-49,948 (71 bp) STS: 49,832-50,007
exon17: 51, 603–51,740 (138 bp)R972G: 51,715(A → G) rs76534745: 43,619,231	5′-CTCTGATGGGAGTGGCTTGG-3′5′-CCACTCAGGCACCCCTTAAC-3′	Chr10: 43,618,871-43,619,601 (713 bp) intron: 51,355-51, 602&51,741-52,085 exon17: 51, 603–51,740 (138 bp)
2q35		
*DIRC3* (noncoding RNA):218,148,746-218,621,316(472571 bp)rs966423:218,310,340	5′-CGGCCTCGACCAACACTTAT-3′ 5′-ACTGGGCGTCTCAACTACAATCTG -3′	Chr2: 218,310,115-218,310,537(423 bp) located in the intron region of DIRC3,
5q24		
*Pre-miR-146a*: 159,912,359-159,912,457(99 bp) rs2910164: 159,912,418	5′-ATTTTACAGGGCTGGGACAG-3′ 5′-TCTTCCAAGCTCTTCAGCAG-3′	Chr5: 159,912,297-159,912,523(227 bp)
8q24		
rs4733616: 128,662,095	5′-CACCGGGGATTGGAAGAGATAAG-3′ 5′- TGAAGCCACAGGGGAGAAAAGT -3′	Chr8:128,661,750-128,662,159(410 bp)
8p12		
*NRG1* transcript variant *HRG-beta1c*: 31,496,820-32,622,558(1,125,738 bp) rs2439302: 32,432,369	5′-AATGCAAGAATGGCCTAACACAAT-3′ 5′-AACCTGGGGSSSSSTCTGAAGC-3′	Chr8: 32,432,326-32,432,660(334 bp) located in intron of NRG1
9q22.33		
rs965513:100,556,109	5′-CCGGCTTGAGTTCAGGTATGTAGT-3′ 5′-CCAGGCTCAGGTTATGTCTTTGTT-3′	Chr9: 100,555,758-100,556,177(420 bp)
9q22		
*FoxE1*: 100,615,537-100,618,997(3,460 bp)rs1867277: 100,615,914	5′-AGACCAGCTGCAGCCACCCCAACC-3′ 5′-GTCTCGCCGCGCTCTTCCTTCACG-3′	Chr9: 100,615,806-100,616,270(465 bp)located in the STS of FoxE1
14q13.3		
rs944289: 36,649,246	5′-CCAGTGGCCCCGCAGGTT-3′5′-GAAAAGCACGTCTCCCCACAGTCC-3′	Chr14: 36,648,944-36,649,435(492 bp)
rs116909374: 36,738,361	5′-TGTAATGGCAGCTCTTGACCTT-3′ 5′-ACCTTTGATTGCCCTTAGTTTGA-3′	Chr14: 36,738,229-36,738,674(446 bp)

## Results

### The identification for the fMNG with PTC

The histological features of the proband’ papillary carcinoma were shown in Figure 2. The members of the Chinese family were diagnosed with MNG and PTC by different hospitals in China; the affected individuals showed typical MNG or MNG with PTC, bilateral and multicentric nodes. In this Chinese family, there were 2 first-degree blood relatives were diagnosed with bilateral MNG and PTC, 5 second-degree blood relatives including a pair of twin sisters were diagnosed with MNG or PTC; Also among these family members, 2 men (III2 and III3), 35 and 25 years old respectively, were diagnosed with MNG and PTC (Figure 1); As the family members resided in different cities and denied radiation exposure, no other neoplasia syndromes or somatic genetic alterations in the tumor DNA was observed, according to diagnostic criteria of familial MNG with PTC [6], we considered the Chinese family presented hereditary predisposition to PTC.

#### The comparison of the susceptibility loci

In the current study, we investigated the exon 15 of *BRAF*, since several SNPs in the genomic region were reported to contribute to PTC in a Chinese population [8] and the transversions in exon 15 are the common morphotype-specific mutation in adult sporadic PTC. The results were shown in Table 3: the examined *BRAF* sequences involved these susceptibility loci carried no risk alleles and were the same as common TT at *BRAF*^T1799A^ and AA at *BRAF*^A1801G^. No any other genetic mutation was found in the family members.

**Table 3:** Sequences of susceptibility loci in the family members

**Chromosome**	***BRAF *** **at Chr7q34**		***RET *** **at Chr10q11.2: 43,572,517-43,625,799**								
	rs121913364	rs113488022	rs35800403	rs2742243	rs77702891	rs80236571	rs75873440	rs77558292	rs77939446	rs80069458	
Locus	140,453,134 exon15	140,453,136 exon15	43,601,415 intron	43,601,749 intron	43,601,894 exon5	43,601,945 exon5	43,607,621 exon8&9	43,609,069 exon10	43,609,070 exon10	43,609,077 exon10	
Allele	A:germline G:germline somatic A → G missense	A:germline; somatic C:somatic T:germline T → A missense	G/C	T/C	A:germline G:germline G → A missense	A:germline G:germline G → A missense	G:germline T:germline G → T missense	T:germline C:germline T → C missense	G:germline A:germline G → A missense	C:germline G:germline C → G missense	
II1 normal	TT	AA	GC	TC	GG	GG	GG	TT	GG	CC	
II2 MNG	TT	AA	GG	TT	GG	GG	GG	TT	GG	CC	
III2 PTC	TT	AA	GC	TC	GG	GG	GG	TT	GG	CC	
II5 PTC	TT	AA	GG	TT	GG	GG	GG	TT	GG	CC	
II8 MNG	TT	AA	GC	TC	GG	GG	GG	TT	GG	CC	
II10 MNG	TT	AA	GG	TT	GG	GG	GG	TT	GG	CC	
Chromosome	*RET* at Chr10q11.2: 43,572,517-43,625,799										
	rs76262710	rs79781594	rs77316810	rs77503355	rs79890926	rs121913313	rs2256550	rs75075748	rs78014899	rs75686697	rs75030001
Locus	43,609,096 exon10	43,609,097 exon10	43,609,102 exon10	43,609,103 exon10	43,609,104 exon10	43,609,104 exon10	43,611,865 exon12	43,613,829 exon13	43,613,840 exon13	43,613,868 exon13	43,613,906 exon13
Allele	C:germline G:germline T:germline T → C&T → G missense	C:germline G:germline G → C missense	C:germline T:germline T → C missense	A:germline C:germline G:germline T:germline G → A& G → C& G → T missense	C:germline G:germline C → G missense	not availiable cds-indel	T/C intron	C:germline T:germline T → C missense	A:unkown C:somatic G:germline G → A& G → C cds-synon	A:germline G:germline G → A missense	C:unkown G:germline G → C missense
II1 normal	TT	GG	TT	GG	CC	no del	TC	TT	GG	GG	GG
II2 MNG	TT	GG	TT	GG	CC	no del	TT	TT	GG	GG	GG
III2 PTC	TT	GG	TT	GG	CC	no del	TC	TT	GG	GG	GG
II5 PTC	TT	GG	TT	GG	CC	no del	TT	TT	GG	GG	GG
II8 MNG	TT	GG	TT	GG	CC	no del	TC	TT	GG	GG	GG
II10 MNG	TT	GG	TT	GG	CC	no del	TT	TT	GG	GG	GG
Chromosome	*RET* at Chr10q11.2: 43,572,517-43,625,799										
	rs77724903	rs79658334	rs11238441	new	rs2472737	rs121913306	rs75234356	rs76087194	rs121913309	rs1800863	rs78347871
Locus	43,613,908 exon13	43,614,996 exon14	43,615,382 intron	43,615,404 intron	43,615,505 intron	43,615,567 exon15	43,615,592 exon15	43,615,611 exon15	43,615,613 exon15	43,615,633 exon15	43,617,398 exon16
Allele	A:germline T:germline A → T missense	A:unkown G:germline T:germline G → A &G → T missense	C/T	C/T	G/A	AGC:germline TTT:somatic cds-indel	G:germline T:germline T → G missense	A:germline G:germline G → A missense	not availiable cds-indel	not availiable C/G cds-synon	C:germline G:germline G → C missense
II1 normal	AA	GG	CC	CC	GA	AGC	TT	GG	no del	CC	GG
II2 MNG	AA	GG	CT	CC	GG	AGC	TT	GG	no del	CG	GG
III2 PTC	AA	GG	CC	CC	GA	AGC	TT	GG	no del	CC	GG
II5 PTC	AA	GG	CT	CC	GG	AGC	TT	GG	no del	CG	GG
II8 MNG	AA	GG	CC	CT	GA	AGC	TT	GG	no del	CC	GG
II10 MNG	AA	GG	CT	CT	GG	AGC	TT	GG	no del	CG	GG

The SNP alleles are shown as the reference/variant, referring to NCBI Build 36.3; the common to mutant is showed by “→”; the risk allele is indicated with an asterisk and outlined if presented in the family members, and the different sequences among the family members are shadowed.

We also investigated all the known *RET* susceptibility loci to family thyroid diseases in this Chinese family. Either, no known *RET* susceptibility loci was mutational in the family members. However, it needs to mention that in the genomic regions which we sequenced, the *RET* introns contained certain differences among the family members, such as introns between exon 4 and 5 (rs35800403 & rs2742243), between 11 and 12 (rs2256550), between 14 and 15 (rs11238441 & rs2472737) (Table 3), and also, there was a new C to T heterozygous allele in the upstream of rs111306965 in the genome of memberII8 andII10 by repeatedly sequencing. Additionally, rs1800863 in exon 15 contained variants of synonymous code substitution in the genome of several family members (II2, II5 & II10).

With respect to the other susceptibility loci identified, as shown in Table 3, all the members from the Chinese family had equal sequences in the (1) *DIRC3* susceptibility locus at 2q35, (2) *Pre-miR-146a* susceptibility locus at 5q24, (3) *NRG1* transcript variant *HRG-beta1c* susceptibility locus at 8p12, (4) susceptibility loci of 8q24, and (5) susceptibility loci of 9q22. Noticeably, all the family members including the proband’s father without thyroid disease were homozygous for the risk alleles of (1) rs966423 (CC) in *DIRC3*, (2) rs2910164 (CC) in *Pre-miR-146a* and (3) rs2439302 (GG) in *HRG-beta1c*; While all these members from the Chinese family contained no risk allele of (4) rs4733616 at 8q24, (5) rs965513 and rs1867277 at 9q22.

For the susceptibility loci of 14q13.3, as 14q was reported to be specifically linked with MNG1 and a form of MNG with PTC [25,26], it is worth to mention that the risk T allele of rs944289 was presented in the sequences of the most family members affected with thyroid disease (II2 & II8, MNG; II5 & III2, MNG with PTC; Table 1). The sequence result in Table 3 showed that both MNG with PTC family members II5 and III2 were heterozygous (CT) and the 2 MNG family members II2 and II10 were homozygous (TT) at rs944289 locus (Table 3). While for another susceptibility locus of rs116909674 at 14q13.3 which we checked, none of the studied Chinese family members carried the risk alleles.

## Discussion

The Chinese family presented hereditary predisposition to PTC, but currently the genetic incline to fPTC is unknown. With the aim of understanding the involvement of genetic factors underlying fPTC, we analyzed the reported possible PTC susceptibility genetic regions by sequence in the Chinese family members who consented to join the study. First, it is worthy to mention that no risk allele of rs965513 (A) or rs1867277 (A) at 9q22 was observed among the Chinese family members. These susceptibility loci of *FOXE1* at 9q22 were related to radiation-induced PTC [19], hence it may be reasonable that the *FOXE1* risk alleles were not presented in the familial form of MNG with PTC, as the members denied radiation exposure and resided in quite different environment. Either, the Chinese family members carried no risk allele of rs4733616 (T) at 8q24 which has been shown to be associated with sporadic PTC in Europeans [14-16], but the pathogenic role of the allele is currently unknown. Our results verified that, for the predisposition to familial form of PTC and radiation-related PTC, their mechanism of PTC susceptibility did not completely overlap each other, since the genetic determinants associated with radiation-related PTC were not presented in the Chinese family members with PTC and MNG.

It is also noticeable that all the family members were homozygous for the risk alleles of rs966423 (CC) at 2q35, rs2910164 (CC) at 5q24 and rs2439302 (GG) at 8p12. All these susceptibility loci have been reported to associate with sporadic PTC [11,13], but currently the pathogenic functions of these alleles are not known well. We think all these risk alleles might contribute jointly to the development of MNG and PTC in the Chinese family members; while considering the risk alleles also presented in the proband’s father with normal thyroid, it is possible that different pathogenic mechanisms exist to activate the tumor transformation in the family members with thyroid disease.

Interestingly, we observed that the frequency of T risk allele of rs944289 at 14q13.3 locus was increased in these MNG and PTC Chinese family members (C: T = 0.4:0.6 vs 0.571:0.429 in normal people). Several studies suggested the possible genetic predisposition of 14q to familial PTC [25] while no association between the radiation-related PTC and 14q13.3 [19]. Also, family nontoxic MNG locus maps to chromosome 14q [24]. Further research suggested that rs944289 was located in a CEBP-alpha/CEBP-beta binding element in the 5-prime UTR of a thyroid-specific lincRNA gene, papillary thyroid carcinoma susceptibility candidate 3 (*PTCSC3*), *PTCSC3* had the characteristics of a tumor suppressor, the rs944289 T risk allele reduced *PTCSC3* promoter activation and thereby predisposes to PTC [21]. Nevertheless, the tumor suppression mechanism of *PTCSC3* is currently unknown. In addition, the thyroid transcription factor of NK2 homeobox 1, *NKX2-1*, is also located in the 14q13.3; *NKX2-1* regulates the expression of thyroid-specific genes involved in morphogenesis. But how rs944289 was associated with *NKX2-1* remains to be investigated. Also, we investigated PTC susceptibility locus of rs116909374 (T) locating between *PTCSC3* and *NKX2-1* at the same 14q, the family members carried no risk allele at all. Hence, our current work implied the possible role of rs944289 in familial MNG with PTC. Whereas, it is surprise that heterozygosity as CT rather than homozygosity as TT presented in the fPTC family members; the same phenomenon was once suggested as a possible special form of genetic epistasis in the rs2910164 allele of *pre-miR-146a* gene [12], which may also contributed to this Chinese fMNG with PTC as shown by the study. Briefly, our results in the Chinese family agreed that rs944289 but not rs116909374 at 14q13.3 locus might be associated with genetic predisposition to familial form of MNG with PTC; it will be intriguing to further analyze the pathogenic link between rs944289 and the disease.

As we failed to detect somatic genetic alterations in the tumor DNA, such as the *BRAF* and *RET* proto-oncogene in the Chinese family members, in the current study, we investigated the genomic region containing the *BRAF* susceptible variants in sporadic PTC, and also all the known *RET* susceptibility loci to thyroid diseases (Tables 2 and 3). Our sequencing results confirmed that the *BRAF* and *RET* mutations were not germline mutations or susceptibility genetic events in this Chinese family. However, we noticed that in the sequenced *RET* genetic region, several different heterozygous alleles were presented among the Chinese family members, and most alleles were in the intron region. Recently, the chromosomal fragile sites breakage was proposed to cause PTC by forming chromosome rearrangement [26]. The chromosomal fragile sites are regions of the genome with a high susceptibility to forming DNA breaks and are often associated with cancer. Exposure to a variety of external factors such as chemotherapeutic, dietary and environmental compounds can induce and accelerate the fragile site breakage. Several intron regions of *RET* were identified as DNA breakage region. Hence, we are wondering if it is possible that the polymorphisms of introns could link to the structural difference in the *RET* region and could impact the related chromosome architecture and thyroid gene expression, albeit there was no *RET* mutation in the cancerous thyroid. Interestingly, there were 2 related facts to be considered: (1) it was shown that transfecting thyroid cells with *RET* produced morphological changes in nuclei that mimicked those seen in PTC [27]; (2) it is curious that the *RET* gene is not expressed in the thyroid follicular cells from which PTC develops, but rearrangements of the *RET* are found in PTC cases [28]. Hence, we think it will be intriguing to investigate the association between the genomic structural of *RET* region and the regulation mechanism of *RET*.

Our work may provide additional evidence to the genetic predisposition to familial form of MNG with PTC. Due to unavailability of samples and the complex of pathogenesis, the current studied Chinese family was small and limited. Nonetheless, for complex diseases like PTC, there may be many genes influencing risk as well as the effects of environment, also, it is much more difficult to collect pedigrees with multiple affected relatives and there is no guarantee of the same (or any) gene (SNP) segregating in these family. To provide insights into the genetic risk factors for familiar PTC, more researches are needed.

## Conclusions

Based on our current investigation in the Chinese fMNG with PTC, the risk allele homozygote of rs966423 (CC) at 2q35, rs2910164 (CC) at 5q24 and rs2439302 (GG) at 8p12 could contribute to the fMNG with PTC, while the other identified risk alleles for sporadic PTC or radiation-related PTC might not be involved. Also, corresponding to the previous studies on the association between chromosome 14q and fMNG with PTC, our work approved that rs944289 but not rs116909374 at 14q13 locus might be associated with genetic predisposition to a Chinese family MNG with PTC. Though several different heterozygous alleles in the *RET* introns presented, the common *BRAF* and *RET* mutations were not susceptibility genetic events in this Chinese family.

## Competing interests

The authors have non-financial competing interests.

## Authors’ contributions

SY L, YQ L and WZ S designed the molecular genetic studies, participated in the sequence alignment and drafted the manuscript. DD D and G X carried out the immunohistochemical assay. SP D and YM L have been involved in revising the manuscript critically. ZL T, JY H participated in data acquisition and helped to draft the manuscript. All authors read and approved the final manuscript.

## Pre-publication history

The pre-publication history for this paper can be accessed here:

http://www.biomedcentral.com/1472-6823/13/48/prepub
